# From the Emergency Department, Directly to Ablation of Atrial Fibrillation: Rationale and Design of the EMERGE Cryo Study

**DOI:** 10.1016/j.cjco.2025.10.019

**Published:** 2025-12-04

**Authors:** Melanie A. Gunawardene, Nele Gessler, Peter Wohlmuth, Daniel Steven, Lars Eckardt, Boris A. Hoffmann, Andreas Metzner, Christian-Hendrik Heeger, Malte Kuniss, Joachim R. Ehrlich, Abdul Shokor Parwani, Philipp Bengel, Claudia Kalkowski, Stephan Willems

**Affiliations:** a Department of Cardiology and Intensive Care Medicine, Asklepios Hospital St. Georg, Hamburg, Germany; b Department of Cardiology, University Hospital Giessen, Giessen, Germany; c MVZ CCB Frankfurt, Frankfurt am Main, Germany; d Semmelweis University, Budapest, Hungary; e Asklepios Proresearch, Hamburg, Germany; f Department of Electrophysiology, University of Cologne, Cologne, Germany; g Division of Electrophysiology, Department of Cardiovascular Medicine, University of Muenster, Muenster, Germany; h Department of Cardiology, Department of Rhythmology, Asklepios Hospital Harburg, Hamburg, Germany; i Department of Cardiology, University Heart Center Hamburg, University Hospital Eppendorf, Hamburg, Germany; j Department of Cardiology, Asklepios Hospital Altona, Hamburg, Germany; k Department of Rhythmology, UKSH Campus Lübeck, Lübeck, Germany; l Department of Cardiology, Kerckhoff-Hospital GmbH, Bad Nauheim, Germany; m Department of Cardiology, St. Josefs-Hospital Wiesbaden, Wiesbaden, Germany; n Department of Electrophysiology, German Heart Center Charite Berlin, Berlin, Germany; o AFNET (Atrial Fibrillation NETwork E.V.), Muenster, Germany

**Keywords:** recent-onset atrial fibrillation, new-onset atrial fibrillation, atrial fibrillation, catheter ablation, emergency department, early rhythm control, cryoballoon

## Abstract

Evidence is limited on an early treatment strategy for any type of recent-onset atrial fibrillation (AF), comparing anti-arrhythmic drug (AAD) therapy to catheter ablation of patients presenting to the emergency department (ED). The purpose of the EMERGE Cryo study is to investigate the impact of early catheter ablation in patients presenting to the ED with recent-onset AF.

The EMERGE Cryo study is a prospective, 2-arm, randomized, open-label, blinded endpoint, multicentre study to investigate the impact of first-line ablation vs AAD in patients presenting to the ED (within the preceding 2 weeks) with recent-onset (≤ 1 year) paroxysmal or persistent AF (longest AF episode < 6-month duration). All study participants receive an implantable loop recorder. Randomization is based on a 1:1 ratio into 2 study arms: (i) cryo-AF-ablation: pulmonary vein isolation performed with cryoballoon; and (ii) usual care: AAD therapy, including electrical cardioversion, if necessary. A total of 350 patients will be randomized.

Both arms have a blanking period or an AAD optimization period of 3 months, during which adapting the therapy will be allowed. This trial is designed to elaborate whether early AF ablation is superior to usual care. The primary effectiveness endpoint is freedom from any atrial tachyarrhythmia (> 30 seconds) through 3-12 months of follow-up on implantable loop recorder monitoring. Patients will be followed up for 36 months in total.

EMERGE Cryo aims to determine the value of early catheter ablation in AF patients presenting with any type of AF to the ED.

**Clinical Trial Registration:**

NCT05294445.

## Résumé

Les données disponibles concernant une stratégie de traitement précoce de tout type de fibrillation auriculaire (FA) d'apparition récente, comparant le traitement par antiarythmiques (TAA) à l'ablation par cathéter chez les patients se présentant aux urgences, sont limitées.

L'étude EMERGE Cryo a pour objectif d'évaluer l'impact de l'ablation précoce par cathéter chez les patients se présentant aux urgences avec une FA récente.

L'étude EMERGE Cryo est une étude prospective, à deux bras, randomisée, ouverte, à critère d'évaluation en aveugle, multicentrique, visant à étudier l'impact de l'ablation de première intention par rapport aux TAA chez les patients se présentant aux urgences (au cours des deux dernières semaines) avec une FA paroxystique ou persistante récente (≤ 1 an) (épisode de FA le plus long < 6 mois). Tous les participants à l'étude reçoivent un moniteur cardiaque implantable (MCI). La randomisation est basée sur un rapport 1:1 dans deux bras de l'étude : (1) cryo-ablation de la FA : isolation des veines pulmonaires réalisée avec un cryoballon et (2) soins habituels : traitement par TAA, y compris une cardioversion électrique, si nécessaire. Au total, 350 patients seront randomisés.

Les deux groupes bénéficient d'une période de consolidation ou d'optimisation des TAA de 3 mois pendant laquelle il sera possible d'adapter le traitement. Cet essai vise à déterminer si l'ablation précoce de la FA est supérieure aux soins usuels. Le critère d'efficacité principal est l'absence de toute tachyarythmie atriale (> 30 secondes) pendant une période de suivi de 3 à 12 mois sous surveillance par MCI. Les patients seront suivis pendant 36 mois au total.

EMERGE Cryo vise à déterminer l'intérêt d'une ablation précoce par cathéter chez les patients atteints de toute forme de FA se présentant aux urgences.

Atrial fibrillation (AF) is the most frequent cardiac arrhythmia in adults,^1^ and it is associated with increased mortality and morbidity. The occurrence of AF is related to a wide variation of symptoms and is a common reason for patients to present to emergency departments (EDs), leading to hospitalizations and high healthcare costs.^2^^,^^3^ Approximately 70% of the patients who are hospitalized for AF are admitted via the ED.^4^^,^^5^ The steady increase of AF-related visits to EDs thereby leads to a high number of hospitalizations.^1^

Catheter ablation therapy has been proven to be safe and effective for the treatment of symptomatic paroxysmal AF and is now the standard for AF therapy.^6^, ^7^, ^8^ Several trials have shown that catheter ablation in this setting is superior to antiarrhythmic drug (AAD) therapy in maintaining sinus rhythm.^9^^,^^10^ As evidenced by the Cryoballon or Radiofrequency Ablation for Paroxysmal Atrial Fibrillation (FIRE & ICE trial), cryoballoon ablation is noninferior to the former gold standard of radiofrequency current energy ablation.^11^ An important finding is that cryoballoon ablation is associateed with a reduction in resource use.^12^

Data on the optimal timing of AF ablation are scarce. Recently, early rhythm control of AF has been shown to be beneficial in preventing negative cardiovascular outcomes.^13^ In a subanalysis of the Early Treatment of Atrial Fibrillation for Stroke Prevention (EAST-AFNET-4) study, symptomatic and asymptomatic recent-onset AF patients derived equal benefit from early rhythm control.^14^

Although catheter ablation has proven to be effective in delaying progression from paroxysmal to persistent AF,^15^ only a few trials have evaluated early AF treatment strategies in relation to patients' medical histories (cryoablation vs. antiarrhythmic drugs: first-line therapy for patients with paroxysmal atrial fibrillation [CRYO-FIRST],^16^ Cryoablation or Drug Therapy for Initial Treatment of Atrial Fibrillation [EARLY-AF],^17^ Cryoballoon Ablation as Initial Therapy for Atrial Fibrillation [STOP-AF FIRST]^18^). Another study examined the implementation of a multidisciplinary AF treatment pathway in ED patients, demonstrating reduced admission rates and hospital stays; however, this approach did not include catheter ablation.^5^

To date, no robust scientific evidence has been gathered from large-scale studies comparing early catheter ablation vs antiarrhythmic drug therapy in AF patients presenting to the ED with recent-onset AF, including those experiencing a first episode. Additionally, the impact of catheter ablation in patients with persistent AF has not been investigated in sufficient detail, as reflected by the current guideline recommendation.^1^

Given that the relevance of AF burden as a study endpoint has gained increasing clinical awareness, continuous follow-up via an implantable loop recorder is considered an essential component of current AF study designs.^19^^,^^20^

Therefore, the purpose of the From the Emergency Department, Directly to Ablation of Atrial Fibrillation (EMERGE Cryo) study is to evaluate the efficacy and safety of an early rhythm control treatment of recent-onset AF by catheter ablation with the cryoballoon vs usual care (AAD), with respect to arrhythmia recurrence, rehospitalization, and heart failure, in patients presenting to the ED or as an emergency visit in an outpatient clinic.

## Methods

### Study design

The EMERGE Cryo study is an investigator-initiated, prospective, 2-arm, randomized, open-label, blinded endpoint, multicentre study to investigate the impact of first-line ablation in patients presenting to the ED or as an emergency visit in an outpatient clinic with recent-onset paroxysmal or persistent AF, including patients with a first episode of AF. According to the statistical analysis plan, n = 350 patients will be enrolled at up to 13 German centres. Each site will contribute < 50% of subjects.

This study has been approved by regional ethics committees and is conducted in accordance with the Declaration of Helsinki and the International Council for Harmonisation Good Clinical Practice guidelines. Written informed consent was obtained from all patients. The trial is registered on ClinicalTrials.gov (NCT05294445).

The principal investigator (PI), and Asklepios Proresearch Institute was responsible for the development of the study protocol. The trial is supported by a steering committee. An independent data and safety monitoring board guides the trial. All serious adverse events and study endpoints are assessed by a blinded core lab and adjudicated by an independent endpoint review committee (ERC), which will be blinded to the randomization group.

The contract research organization RQM+ provides support with regard to monitoring of patient data, site supervision, site initiation, and closeout visits. The contract research organization and the study sites use secuTrial (interActive Systems GmbH, Berlin, Germany) as the electronic case-report form system.

### Endpoints

The *primary effectiveness endpoint* is freedom from any atrial tachyarrhythmia, including AF, atrial flutter, and atrial tachycardias (> 30 seconds [s]) through 3 to 12 months of follow-up on implantable loop recorder [ILR] monitoring, or any 12-lead electrocardiogram [ECG] on visits, ECG Holter monitoring, or on symptom-driven event monitoring).

This trial is designed to demonstrate that early AF ablation is superior to usual care in patients presenting to the ED because of AF, with respect to clinical treatment success, which is defined as freedom from atrial arrhythmias (> 30 seconds) through 3-12 months of follow-up care.

Both arms will have a blanking period or an AAD optimization period of 3 months, during which therapy adaptation will be allowed. A repeat ablation procedure will not reset the blanking period. AF occurring during the blanking period, as well as any interventions started during the blanking period, do not count as chronic treatment failures. Study endpoints will be adjudicated by a blinded core laboratory responsible for ILR data analysis, in conjunction with the study's independent ERC. Safety outcomes are not subject to any blanking.

### Secondary effectiveness endpoints of the EMERGE Cryo study are as follows:

• AF burden between 3-12 months after randomization; AF burden is defined as the percentage of time the study participant is in AF during the observation period;
• AF burden between 0-3, 3-6, and 6-12 months after randomization;
• freedom from AF (> 30 seconds) through 3-12 months of follow-up care;
• freedom from atrial tachycardia and atrial flutter (> 30 seconds) through 3-12 months of follow-up care;
• analysis of amount of symptomatic vs asymptomatic atrial tachyarrhythmia recurrences through 3-12 months of follow-up care (reported as number of recurrence episodes);
• rehospitalization rate due to cardiovascular disease (AF, worsening of heart failure, cardiovascular disease);
• progression of heart failure defined as trend in left ventricular ejection fraction and trend in brain natriuretic protein;
• improvement of quality of life (QoL) at 12 months compared to baseline (**A**trial **F**ibrillation **E**ffect on **Q**uali**t**y-of-life [AFEQT] questionnaire and EuroQol 5-dimension, 5-level [EQ-5D-5L] questionnaire); and
• safety / complications: device- and procedure-related adverse events, serious adverse events, and cardiovascular-related adverse events will be collected to determine and compare the safety profile of the 2 treatment groups.

### Study setting and timeline

As of August 15th, 2025, a total of 13 sites in Germany have been activated or have pending activation, with a total of 319 patients enrolled. We aim to complete enrollment of 350 patients within 4 months (by October 2025). Appendix Material S1 and S2.

### Patient population

Inclusion criteria include recent-onset (≤ 1 year prior to enrollment) documented paroxysmal or persistent AF (longest AF episode < 6-month duration), presenting at the ED or an outpatient clinic as an emergency visit within the preceding 2 weeks because of AF, including patients with spontaneous conversion to sinus rhythm (with prior AF documentation). This group also includes patients presenting with their first clinical episode of AF, and patients with both symptomatic and asymptomatic AF. Patients meeting the study inclusion and exclusion criteria as listed in Table 1 will be screened, by the investigator or the investigator’s designee, from the investigator’s general patient population. Patients who meet the enrollment criteria and sign the informed consent form will be included in the study.

**Table 1:** Inclusion and exclusion criteria

Inclusion criteria:
• documented paroxysmal or persistent AF (longest AF episode < 6-month duration). Any ECG documentation of AF (12-lead ECG, Holter ECG, or mobile ECG monitoring) needs to be presented. • recent-onset AF (≤ 1 year prior to enrollment) • presenting at the emergency department or outpatient clinic within the last 2 weeks because of AF, including patients with spontaneous conversion in sinus rhythm (with prior AF documentation) • age ≥ 18 years • subject is able and willing to give informed consent
Exclusion criteria:
• persistent AF > 6 mo (1 episode) • LA diameter > 60 mm • severe mitral stenosis or regurgitation, prior mitral valve reconstruction or replacement • any previous LA ablation • ongoing continuous AAD therapy with amiodarone at baseline • history of failed continuous AAD therapy with > 1 agent. Exceptions are beta-blocker, verapamil, or “pill in the pocket” therapy • any condition or disease, which is a contraindication for AF ablation, per the assessment of the investigator • any condition or disease, which is a contraindication for AAD treatment, per the assessment of the investigator • known intracardiac thrombus formation under continuous oral anticoagulation (defined as intake > 4 wk) • any contraindication for oral anticoagulation • any untreated or uncontrolled hyperthyroidism or other reversible causes for AF, such as alcoholism • pregnant or breastfeeding woman or woman of childbearing potential not on adequate birth control • active systemic infection • coexistence of non-PV-dependent atrial tachycardia∗

AAD, anti-arrhythmic drug; AF, atrial fibrillation; ECG, electrocardiogram; LA, left atrial; PV, pulmonary vein.

∗ This includes any prior ECG of focal atrial tachycardia, atypical atrial flutter, and/or atrioventricular (node) re-entrant tachycardia.

### Trial flow

At baseline, an ECG, a transthoracic echocardiogram, a QoL questionnaire (EQ-5D-5L and AFEQT), as well as certain blood tests will be performed for every patient. Randomization takes place at baseline. Patients who are in the intervention group will receive cryoballoon-guided AF ablation within 21 days from baseline. Patients who are in the control group will receive standard-of-care only, which is defined as a rhythm control strategy with electric cardioversion (if necessary) and AAD. Both arms will receive an ILR for continuous rhythm monitoring. Regular follow-up evaluation is planned for both groups after 3, 6, and 12 months. An extended follow-up evaluation of the ILR events up to 24 and 36 months is planned, including a patient phone call at 24 and 36 months for detection of adverse events, and QoL assessment (sent via letter).

**Figure 1:**
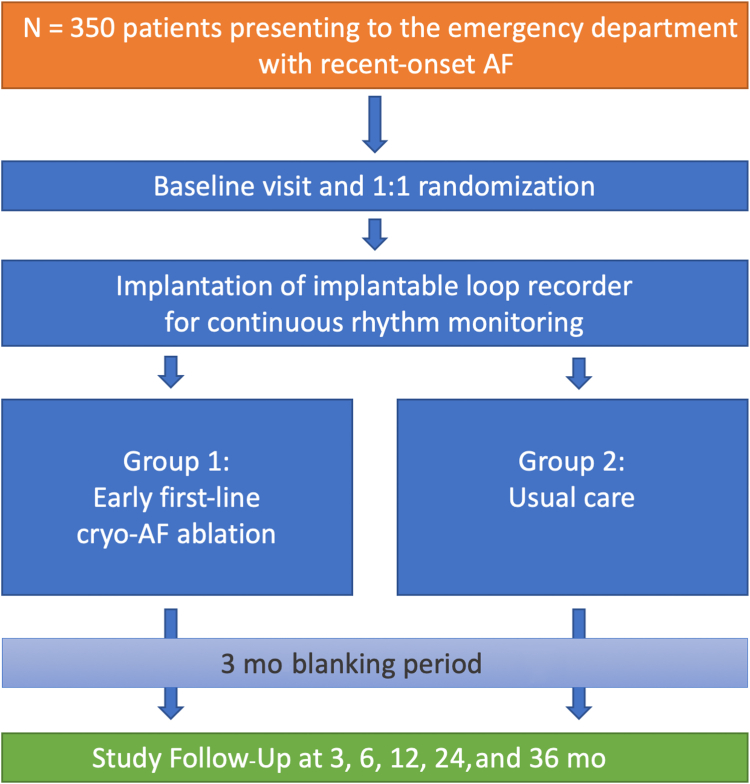
Study flowchart. AF, atrial fibrillation.

Randomization is based on a 1:1 ratio into 2 study arms, as follows: (i) cryo AF ablation—pulmonary vein isolation (PVI) performed with a cryoballoon ablation system; and (ii) usual care—AAD therapy based on the decision of the investigator according to current European Society of Cardiology (ESC) guidelines,^1^ including electrical cardioversion, if necessary. Both arms will have a blanking period or an AAD optimization period of 3 months, during which adaptation of the therapy will be allows (Fig. 1).

### Data collection within the study

Data collection within the study, including baseline testing, is reported in Table 2.

**Table 2:** Data collection within the study

Data collection	Baseline	ILR-Implantation	Ablation	Pre-discharge	3-mo	6-mo	12-mo	24-mo	36-mo
(d 0 = presenting in ER with recent-onset AF)	(0–21 d, prior to ILR-implantation)	(0–21 d, prior to ablation / AAT)	(0–21 d) (Ablation group)	(Ablation group)	(+/- 7 da)	(+/- 7 d)	(+/- 7 d)	(+/- 14 d)	(+/- 36 d)
Informed consent	x								
Inclusion / exclusion	x								
Demographic data	x								
Medical history	x								
Medication regime (cardiovascular)	x		x	x	x	x	x		
Randomization	x								
Procedural data (only ablation group)			x						
ECG	x†				x	x	x		
48 h Holter∗									
TTE	x‡					x	x		
(LAvolume, LVEF)									
Questionnaires:	x						x	x	x
AFEQT									
EQ-5D-5L									
AF symptoms	x				x	x	x		
EHRA Score; patients’ diary									
Blood tests (creatinine, glomerular filtration rate, BNP, potassium, thyroidal-stimulating hormone, blood count incl. hemoglobin, GOT, GPT, GGT, INR)	x§					x	x		
ILR interrogation		x			x	x	x	x	x
TEE (only intervention group)			x						
Physical examination	x				x	x	x		
Adverse event reporting	x		x	x	x	x	x	x	x
Phone call								x	x

AAT, antiarrhythmic drug therapy; AF, atrial fibrillation; BNP, brain natriuretic protein; EHRA, European Heart Rhythm Association; EQ-5D-5L, EuroQol 5-dimension, 5-level; EQT, **A**trial **F**ibrillation **E**ffect on **Q**uali**t**y-of-life; ER, emergency room; GGT, gamma-glutamyl transferase; GOT, glutamic-oxaloacetic transaminase; GPT, glutamate-pyruvate transaminase; ILR, implantable loop recorder; incl, including; INR, international normalized ratio; LA, left atrial; LVEF, left ventricular ejection fraction; TEE, transesophageal echocardiography; TTE, transthoracic echocardiography.

∗ Holter-ECG is recommended, not mandatory.

† ECG performed within clinical routine prior to d 0 may be used.

‡ TTE performed within clinical routine prior to d 0 may be used.

§ Blood sample performed within clinical routine prior to d 0 may be used.

### Implantable loop recorder

All study participants will receive an ILR (Reveal LINQ, Medtronic, MN) within 21 days of the baseline visit, and no later than the date of therapy initiation (ie, the cryoballoon ablation procedure or the start of antiarrhythmic drug treatment).

Patients in the ablation arm undergo the implantation procedure during their hospital stay for AF ablation. Patients in the usual-care arm undergo the implantation procedure either during their hospital stay at baseline (for inpatients) or within 21 days from baseline (for outpatients). The implantation procedure will be performed according to device instructions, standard of care, and current guidelines.

AF burden (time spent in AF) is collected by the ILR. The ILR provides a daily measure of the time spent in AF. The ILR will be enrolled in the CareLink system (Medtronic) for central blinded remote monitoring (for research only).

A core lab with blinded investigators, assisting the ERC, will assess the detected AF episodes.

### Catheter ablation group

Patients randomized into the cryoballoon ablation group should receive the catheter ablation within 21 days. The decision to perform transesophageal echocardiography prior to ablation is made per the physician’s discretion, according to current guidelines. For the sake of comparability of data, the ablation should be performed with the Arctic Front Advance Cardiac Cryo Ablation Catheter System (Medtronic). If the cryoballoon ablation procedure should fail or be skipped for any reason, the patient will remain in the study and will be followed according to the intention-to treat principle. The ablation strategy of this study group has been described previously.^16^ Regarding the ablation strategy, PVI is the primary ablation target. Conduction of the cryoballoon ablation strategy can be performed at the discretion of the operator. In the case of ongoing AF after PVI, cardioversion is attempted. If cardioversion fails, further ablation may be performed at the discretion of the operator. In the case of concomitant typical atrial flutter, cavotricuspid isthmus ablation should be performed with radiofrequency current.

### Usual care group

Patients randomized into the usual care group should start or be maintained on antiarrhythmic drug therapy within 21 days, based on the decision of the investigator, according to current ESC guidelines.^1^ Usually, class Ic AAD (eg, flecainide, propafenone) will be used apart from patients with comorbidities and/or impaired left ventricular function, who will preferably receive amiodarone. If necessary, cardioversion can be performed to establish sinus rhythm according to current ESC guidelines.^1^ Oral anticoagulation and treatment of comorbidities will be performed at the discretion of the investigator, according to current guidelines, for both study arms.^1^

### Statistical methodology

The study’s statistical analysis plan outlines the following: The primary analysis is related to atrial tachyarrhythmias and AF. A survival table and a Kaplan-Meier plot with stratified survival curves by groups will be shown. A log-rank test will be applied to examine differences of the hazard function between the treatment groups. A *P*-value of < 0.05 will be considered significant.

Secondary analyses are associated with QoL, left ventricular ejection fraction, and brain natriuretic protein over time. (Generalized) linear mixed models will be used to examine differences between ablation groups. AF burden and the rehospitalization rate will be analyzed descriptively. The time to first rehospitalization will be presented as survival tables and plots. Secondary endpoints were considered exploratory.

Procedure-related complications, including the index and any repeat catheter ablation, ILR implantation, and cardioversion, will be observed. As the serious adverse events are captured in free text, they will be harmonized (by the trial steering committee) before analysis. Each single type of adverse event for a subject will be shown only once.

All serious adverse events, and their duration, resolution, cardiac cause, and severity are listed. The summary table comprises only the treatment-related adverse events with a cardiac cause.

The regular study phase concludes after 12 months, including site monitoring as well as ERC and data and safety monitoring board oversight. During the subsequent prolonged follow-up period (up to 24 and 36 months), rhythm-related endpoints via ILR, and serious adverse events, will continue to be collected.

### Sample size calculation

Pokushalov et al.^21^ showed a freedom from AF of 59% in paroxysmal and persistent AF (combined) with continuous intracardiac monitoring after catheter ablation of AF. In the **C**atheter **Ab**lation vs **An**ti-Arrhythmic Drug Therapy for **A**trial Fibrillation (CABANA) trial,^9^ the outcome difference between ablation and antiarrhythmic drugs was 19.6% (49.9% vs 69.5% for AF recurrence). In the meta-analysis from Asad et Al.,^22^ including 18 randomized trials, recurrence from atrial arrhythmias was 36% after catheter ablation, and 67% after AAD therapy, with a risk ratio of 0.42 favouring catheter ablation.

We therefore hypothesize that, in our trial, the outcome of AF free survival will be 60% in the ablation group and 45% or less in the conventional group during 12 months of follow-up care. This study is planned with 80% power to detect a difference between treatment groups at a 2-sided alpha level of 0.05 using a 2-sample log-rank test. With a suspected dropout rate of 10%, a total of 350 patients is required.

### Funding

The EMERGE Cryo study is funded by a research grant for investigator-sponsored trials from Medtronic. The funding source had no role in the design of this study and will have no role in study execution, data collection/analysis or interpretation, writing of the report, or decisions on when and where to submit the report for publication. The study has been endorsed by the AFNET (Atrial Fibrillation NETwork E.V.) without any financial support.

## Discussion

### Rhythm control

Catheter ablation has been shown to be an effective treatment for symptomatic, predominantly paroxysmal AF patients. Three randomized studies included patients with paroxysmal AF who underwent first-line cryoballoon ablation; these studies exclusively found catheter ablation to be superior to AAD in maintaining sinus rhythm.^16^, ^17^, ^18^ However, these trials included a specific, and predefined, cohort of rather young patients with few comorbidities.^16^, ^17^, ^18^ Accordingly, the current ESC guidelines recommend catheter ablation as a first-line treatment option, based on shared decision-making, for patients with symptomatic paroxysmal AF, to reduce symptoms, recurrence, and disease progression.^1^ Due to the sparseness of the data, this recommendation does not include patients with any type of persistent AF, even those with AF of rather short duration—lasting more than 1 week but not months.

In comparison, the EMERGE Cryo study cohort consists of patients with recent-onset AF who present to the ED, irrespective of AF phenotype and symptoms. This group may include patients experiencing their first clinical episode of AF, as well as those with and without symptoms.

Thus far, asymptomatic patients have not been enrolled systematically in randomized trials investigating catheter ablation. Especially with the emergence of wearables, more people may detect cardiac arrhythmias through their devices and present to outpatient clinics or ED with incidental diagnosis of AF.^23^ Rhythm control treatment of asymptomatic AF is not well understood. As mentioned, a subanalysis from the EAST-AFNET-4 study showed that early rhythm control may be beneficial in asymptomatic AF patients as well.^14^ Given that patients are randomized to receive either AAD ± cardioversion, or cryoballoon ablation, including all patients presenting with recent-onset AF is both appropriate and clinically relevant. This approach allows the EMERGE Cryo study to assess long-term outcomes in a broader population and has the potential to provide novel insights into the determination of which subgroups derive the greatest benefit from an early invasive strategy.

Additionally, data on optimal treatment of the first clinical episode of AF are lacking. A first detected episode of AF appears to be a marker for underlying cardiac diseases.^24^ Moreover, recent-onset AF is associated with an increased likelihood of mortality.^25^ Among patients who have transient new-onset AF during a hospitalization for noncardiac surgery or medical illness, approximately 30% had recurrent AF within 1 year.^26^ Although rhythm-control strategies for recent-onset AF, including a first episode of AF, are not well understood, a shorter time between first AF diagnosis and ablation is linked to higher procedural success.^27^ Therefore, the time between ED presentation and rhythm control (either cryoablation or AAD use) has been set to a maximum of 21 days in the EMERGE Cryo study. This timeframe may also allow for early intervention in AF treatment, even in patients presenting with a first episode.

One limitation of the study is the potential clinical heterogeneity inherent to an ED-based AF population. Although exclusion criteria were applied to eliminate patients with transient or reversible causes of AF (eg, systemic infection, hyperthyroidism, or acute alcohol use), the inclusion of both symptomatic and asymptomatic presentations, along with a range of AF types (including first-episode, paroxysmal, and short-duration persistent AF), may result in a more heterogeneous study population that could influence treatment response and clinical outcomes. Although the study population includes patients with varying clinical presentations, all patients share two key unifying characteristics—recent-onset of AF (< 1 year) and presentation to an emergency or urgent care setting. This commonality reflects a clinically relevant and underrepresented subgroup not specifically addressed in previous randomized trials. Although this subgroup may introduce some variability in treatment response, this real-world diversity reflects the complexity of AF management in the acute care setting and may enhance the generalizability of the findings.

In the EMERGE Cryo study, we aim to challenge current paradigms regarding the timing of catheter ablation by exploring whether early intervention can be extended to a broader patient population—including asymptomatic individuals, those with persistent AF, and those experiencing their first AF episode—who present to the ED. At the same time, the study establishes a short, standardized interval between ED presentation and initiation of rhythm control.

### ILR

A well-known limitation of many trials investigating catheter ablation of AF is the sporadic and/or occasional ECG follow-up after ablation, as detection of AF recurrences can be challenging^28^ and depends on the monitoring strategy.^19^ The sensitivity of detection of asymptomatic AF episodes with intermittent 24-hour ECG-monitoring is low,^29^ which is a strong limitation, as a well described finding is that symptomatic AF may convert to the asymptomatic form.^30^ Thus, the Heart Rhythm Society and the European Heart Rhythm Association encourage continuous arrhythmia monitoring due to its greater sensitivity in not only detecting symptomatic and asymptomatic AF recurrences but also assessing the overall AF burden.^6^^,^^29^^,^^31^ Additionally, in an era of digital revolution, the AFNET incorporated the use of wearables, smartphones, hand held-devices, and health-related apps into new approaches to AF management.^32^ The recently published Pulsed Field Ablation of Persistent Atrial Fibrillation With Continuous ECG Monitoring Follow-Up (ADVANTAGE phase II) study, in which persistent AF patients underwent pulsed field ablation-guided catheter ablation, a postablation AF burden of < 0.1% was associated with the lowest healthcare utilization.^20^

The occurrence of ≥ 30 seconds of atrial arrhythmia was selected as the primary endpoint, in accordance with established standards at the time of study design, and remains the most commonly reported arrhythmia endpoint in recent landmark trials.^33^^,^^34^ Nonetheless, additional clinically relevant outcomes, such as AF burden and patient-reported measures, have been included as secondary endpoints to enable a more comprehensive assessment.

Due to the timing of ILR implantation, a comparison of AF burden before and after ablation is not feasible in the EMERGE Cryo study, an acknowledged limitation of the study.

However, we believe that the use of an ILR within the EMERGE Cryo study provides the gold standard and allows for assessment of the true AF burden, and therefore the time spent in AF. Additionally, the EMERGE Cryo study will provide an extended follow-up evaluation of up to 36 months for secondary endpoints. Yet, a limitation of this study—similar to those of other trials in this space—is that it is not powered to detect differences in hard clinical outcomes such as stroke, hospitalization, or mortality, as it is primarily designed to assess AF recurrence.

## Conclusion

The EMERGE Cryo study is an investigator-initiated, prospective, 2-arm, randomized, open-label, blinded endpoint, multi-centre study to investigate the impact of first-line ablation in patients presenting at the ED with recent-onset paroxysmal or persistent AF. The trial is currently ongoing and completion of patient enrollment is planned for fall of 2025. The trial's outcome aims to determine the value of early catheter ablation in AF patients presenting to the ED, irrespective of symptoms, and AF phenotype, and including those with recent-onset AF or their first clinical AF episode. As a result, this study has the potential to address evidence gaps, reduce the AF burden in patients, and lower rehospitalization rates throughout an extended follow-up period.
